# Heart and lung point-of-care ultrasonography tutoring in internal medicine: a randomized controlled trial

**DOI:** 10.1007/s40477-024-00968-8

**Published:** 2024-12-03

**Authors:** Antonio Leidi, Juliette Krauer, Guillaume Soret, Thibault Parent, Christophe Marti, Philippe Meyer, André Juillerat, Stijn Bex, Noémie Suh, Frédéric Rouyer, Nils Siegenthaler, Olivier Grosgurin

**Affiliations:** 1 Division of General Internal Medicine, Department of Medicine, Geneva University Hospitals, Rue Gabrielle-Perret-Gentil 4, 1205 Geneva, Switzerland; 2 Division of Emergency Medicine, Geneva University Hospitals, Geneva, Switzerland; 3 Division of Cardiology, Geneva University Hospitals, Geneva, Switzerland; 4 Division of Intensive Care, Geneva University Hospitals, Geneva, Switzerland; 5 Division of Intensive Care, La Tour Hospital, Geneva, Switzerland

**Keywords:** POCUS, Ultrasound, Internal medicine, Tutoring, Skill retention, Proficiency

## Abstract

**Purpose:**

In recent years, point-of-care ultrasonography (POCUS) has been integrated into internal medicine practice with most curricula composed of a single course. Despite competency acquisition during the course, a significant decline in proficiency occurs in the subsequent weeks due to a lack of regular practice and training. This study aims to evaluate the impact of a structured POCUS tutoring, on POCUS use and skills retention at 6 months.

**Methods:**

In this randomized controlled trial, internal medicine residents were enrolled after completing a practical course focused on heart and lung ultrasonography. Residents assigned to the intervention group were paired with a tutor, and time slots were scheduled for bedside direct supervision over the following 6 months. The primary outcome was the proportion of residents who successfully completed at least 25 POCUS examinations six months after inclusion. Secondary outcomes included self- and blinded-expert-assessed POCUS competency skills.

**Results:**

Between January and May 2022, 23 residents were included in the study. The intervention significantly increased the proportion of participants reaching the primary outcome (70% versus 0%; *p* < 0.001) with a median of 27 (interquartile range, IQR, 6 to 44) supervised examinations performed versus 0 (IQR 0 to 0) in intervention and control arm respectively; *p* < 0.001. After 6 months, proficiency was higher for most heart imaging but not for lung imaging, as assessed through self-assessment by participants or independently by blinded experts.

**Conclusion:**

Structured POCUS supervision significantly enhances the number of POCUS examinations and proficiency at 6-months, particularly in applications requiring greater visuospatial skills.

**Supplementary Information:**

The online version contains supplementary material available at 10.1007/s40477-024-00968-8.

## Introduction

In recent years, point-of-care ultrasonography (POCUS) has emerged as a useful tool in clinical practice, enabling clinicians to address basic diagnostic questions, guide procedures, and make decisions regarding immediate therapies [1, 2]. The value of POCUS fundamentally relies on the skills of the operator as unthoughtful use may lead to unfounded reassurance, increased numbers of additional testing, incorrect diagnosis, and treatment [3]. For this reason, minimal requirements for POCUS use in internal medicine have been established worldwide [4, 5]. In Switzerland, certification exists for 15 POCUS modules, three of which are particularly relevant in internal medicine: emergency ultrasonography (E-FAST, gallbladder, kidneys and bladder, aorta and venous compression ultrasound), focused echocardiography, and thoracic ultrasonography (US). The modules are comprised of theoretical and practical coursework, followed by practical training through a total of 200 POCUS examinations. Half of these must be supervised by a certified POCUS instructor. In some instances, combining two modules reduces the total amount of required examinations to 100 per module.

Postgraduate curricula have been developed to familiarize young doctors with basic US investigations; most of them are exclusively composed of a theoretical and practical one-day course. While trainees significantly increase their competencies during the course [6], most of them experience a net loss of proficiency after 1–2 years because of lack of practical training [7]. A decline in skills is observed within the first weeks following the course [8] and low engagement in practice is common as only 6% of participants perform at least 30 examinations six months after the course [9]. Lack of training, absence of direct supervision, and insufficient time were the main perceived barriers to learning and using POCUS in previous surveys among North American and European internists [10–12].

The aim of the present study was to investigate whether a structured longitudinal POCUS tutoring, including scheduled time slots for bedside direct supervision, could enhance POCUS use and skills retention among internal medicine residents 6 months after a theoretical and practical course.

## Methods

This study was a single-center randomized open-label superiority trial. Recruitment of participants took place at the general internal medicine service of the Geneva University Hospitals. At our institution, internal medicine residency consists of three years of postgraduate medical practice including several 4-month clinical rotations in external services, such as emergency medicine, intensive care, medical specialties and/or rehabilitation medicine. Residents usually spent two years in secondary hospitals before being integrated in our residency. Since 2019 a POCUS training program has been offered to the residents and at the outset of the study consisted of a pre-course e-learning and a single one-day hands-on supervised practical course focused on heart and lung US. Following this training, and after having obtained written informed consent, residents with little or no experience in POCUS were randomized in two groups with a 1:1 ratio. Participants were allocated by an independent investigator using a computer-generated randomisation list with no stratification nor block permutation. Participants allocated to the intervention arm were assigned to a certified POCUS tutor and time slots were scheduled for direct bedside supervision. Timeslots were agreed upon between trainees and supervisors through an agenda crosscheck; there was no allocation of dedicated time specifically for the purpose of the study. Once the tutor determined that core competencies had been acquired, a web-based indirect asynchronous supervision was allowed through an electronic logbook (LogIC®, Reallience, Switzerland): participants performed POCUS independently and then sent the recorded and interpreted images and/or videos to the supervisors for asynchronous validation.

Participants allocated to the control arm were provided with a list of available tutors and were encouraged to practice supervised POCUS on a voluntary basis but no time slots were scheduled in advance (i.e. standard of practice at our institution). In addition, they had full access the same web-based platform for asynchronous supervision (LogIC®, Reallience, Switzerland). The intervention period started immediately after the practical course and lasted for 6 months. The trial design is schematically represented in Supplementary Fig. 1. The local ethics committee confirmed that the present study was exempt from the requirement for formal approval.

### Primary outcome

The primary outcome was the proportion of residents who successfully completed a total of at least 25 direct- and/or asynchronous- supervised POCUS examinations 6 months after inclusion. Results are presented for the total population and a pre-specified subgroup of clinical rotations. Because of their higher availability of POCUS machines, internal, emergency and intensive medicine rotations were considered as favourable rotations. All other clinical rotations (e.g. immunology, rehabilitation) were considered as unfavourable rotations for POCUS.

### Secondary outcomes

The total number of POCUS examinations, self-assessed POCUS competencies, and expert-assessed proficiency were evaluated as secondary outcomes.

#### Self-assessed competencies

Immediately after the practical course and at the end of the 6-month intervention period, all participants were asked to answer a self-assessment survey (Supplementary Table 1). Global satisfaction, global competencies in POCUS use and specific self-assessed competencies in heart and lung US were evaluated with a 5-point Likert scale (1 = “Strongly disagree” to 3 = “Neutral” to 5 = “Strongly agree”). For the purpose of simplicity, answers were grouped in a dichotomic way, “Satisfied” or “Competent” (i.e. “Strongly agree” to “Agree”) or “Unsatisfied” or “Incompetent” (i.e. “Neutral” to “Strongly disagree”).

#### Blinded expert assessment

Three months after the 6-month intervention period, the performances of participants were evaluated by blinded external POCUS experts (i.e. POCUS certified doctors with more than 5 years of POCUS practice). Participants were asked to perform focused echocardiography (i.e. parasternal views, apical four- and five-chamber views, subcostal 4-chamber view with IVC) and lung US (i.e. parasternal sagittal view and costophrenic recess) on one healthy volunteer and one ambulatory patient with known and documented left ventricular systolic dysfunction. Participants and experts were blinded to the clinical data, experts were masked to the participants’ allocation arm. POCUS competencies were evaluated by experts through an adapted version of previously published assessment tools [13, 14] Global POCUS competencies were evaluated using a 3-point Likert scale (1 = “Some criteria obtained” to 3 = “All criteria obtained”), answers were grouped in “Competent” (i.e. “Most or All criteria obtained”) and Incompetent (“Some criteria obtained”). General autonomy was evaluated by experts using a 5-point Likert scale (1 = “I had to perform the exam” to 5 = “My presence is unnecessary”), answers were grouped in “Autonomous” (i.e. 3 = “Verbal correction from time to time” to 5 = “My presence is unnecessary”) and “Not Autonomous” (i.e. 1 = “I had to perform the exam” to 2 = “I have to talk them through and reposition the probe”). Quality of imaging was rated on a 6-point Likert scale ranging from (0 = “Not obtained” to 3 = “Suboptimal quality but interpretation possible” to 5 = “Good quality, meaningful interpretation easy”), answers were grouped in “Poor quality” (zero to two) and “Good quality” (three to five). Moreover, experts rated in a dichotomic way (yes/no) whether the quality of images allowed a meaningful interpretation of left ventricular systolic function (LVF), right ventricular dilatation (RVD), hypervolemia and pericardial effusion. Participants’ conclusions regarding left ventricular systolic function (i.e., normal, mild to moderate dysfunction, severe dysfunction) were documented and compared to ambulatory echocardiography results from a reference cardiologist, conducted within a maximum of one year prior to the evaluation.

The collected data were subsequently included into the study database with double checking by an independent data service provider (Data Conversion Service SA, Geneva, Switzerland).

### Sample size

Based on previous reports and local experience, we expected that the proportion of success in the control group would not exceed 20% [9] A sample size of 20 residents was calculated to detect a 60% difference in the primary outcome (alpha of 0.05; 0.90 of power). The sample size was increased to 22 participants to anticipate 10% of loss to follow-up.

### Statistical considerations

Baseline participants’ characteristics and characteristics of POCUS examinations are presented with descriptive statistics. Results of the primary outcome are presented in proportions, risk difference (RD), and number needed to supervise (NNS = 1/RD). Differences in the primary outcome are compared with the Fischer exact test for non-parametric distribution. Number of supervised POCUS examinations are compared using the Wilcoxon Mann–Whitney test. Between-group differences in self- and expert-assessed competencies are compared with the Chi-squared or Fischer exact test, as appropriate. Data are visually presented in the form of bar graphs. The proportion of participants drawing the right conclusion on LVF are compared between study arms with the Fischer exact test. A two-sided p-value of 0.05 is used to infer statistical significance. All statistical analyses were performed using a standard software package (Stata, version. 16.1; StataCorp).

## Results

Between January and May 2022, 27 general internal medicine residents were assessed for eligibility; due to extensive prior experience with POCUS, 4 of them were excluded and 23 residents were included in the study; 11 were allocated to the intervention group (55% of women, mean age of 29.6 ± 1.0 years) and 12 to the control group (75% of women, mean age of 29.9 ± 1.6 years). Follow-up ended on the 26th of January 2023. Baseline characteristics are shown in Table 1. Participants had variable clinical experience in internal medicine (two to five years after medical graduation) and small or no previous experience in POCUS. All participants intended to specialize in internal medicine. Characteristics of POCUS tutors are available in Supplementary Table 3.

**Table 1:** Baseline characteristics of participants by study group

	Intervention (*n* = 11)	Control (*n* = 12)
Sex		
Female	6 (55%)	9 (75%)
Male	5 (45%)	3 (25%)
Age in years, mean (SD)	29.6 (1.0)	29.9 (1.6)
Years since medical degree, *n* (%)		
2 years	3 (27%)	1 (8%)
3 years	1 (10%)	3 (25%)
4 years	3 (27%)	3 (25%)
5 years	4 (36%)	5 (42%)
Years since first POCUS use, *n* (%)		
0 years	4 (36%)	5 (42%)
1 year	5 (46%)	7 (58%)
2 years	2 (18%)	0 (0%)
Planned specialisation^a^		
Internal medicine	11 (100%)	12 (100%)
Emergency medicine	1 (9%)	0 (0%)
Infectiology	0 (0%)	1 (8%)
Gastro-enterology	1 (9%)	0 (0%)
Main clinical rotation during study period		
Favourable^b^	4 (36%)	7 (58%)
Unfavourable	7 (64%)	5 (42%)

*SD* standard deviation, *POCUS* point of care ultrasonography

^a^Three participants intended to obtain a double specialisation

^b^Internal, Emergency and Intensive care medicine rounds were considered favourable clinical rotations

**Fig. 1:**
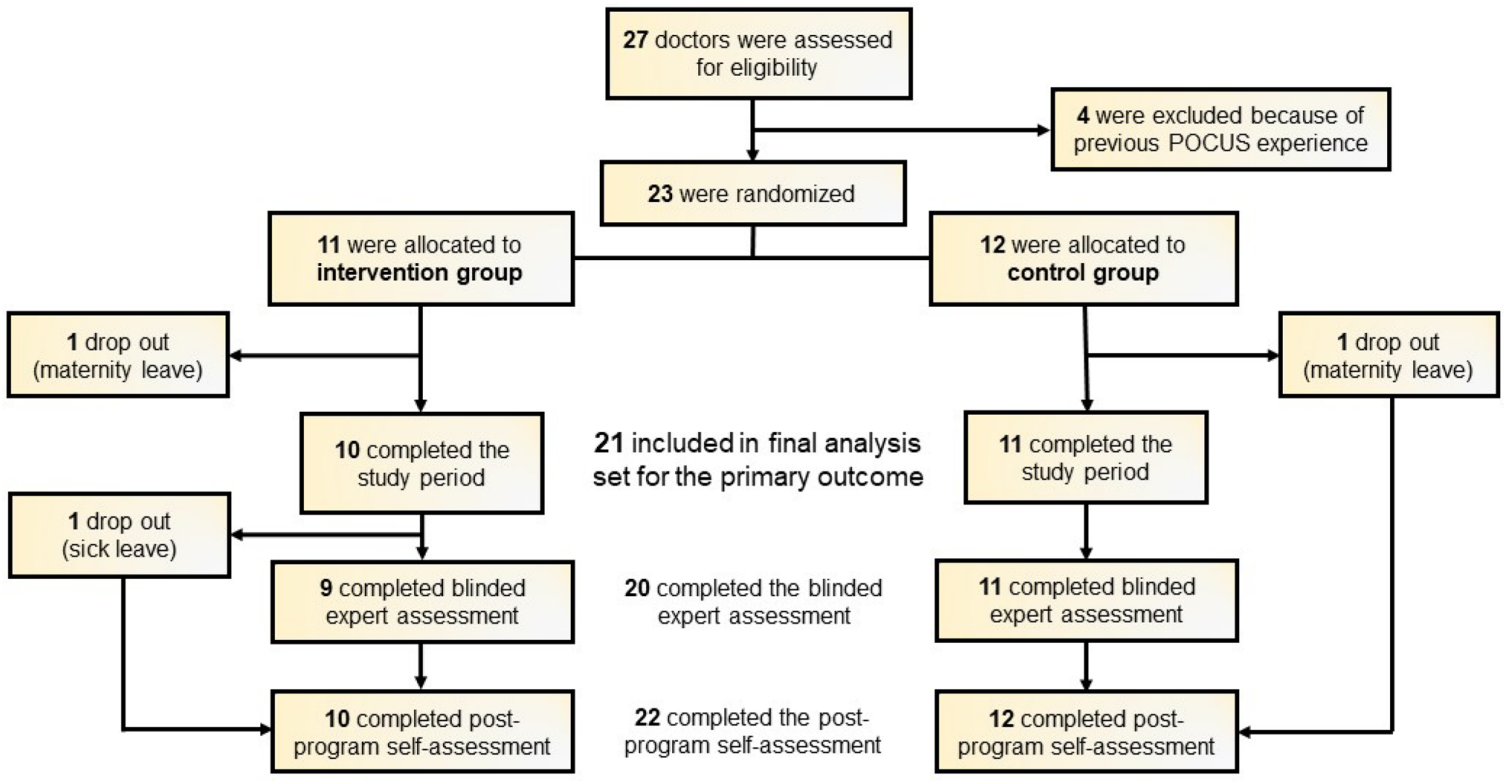
Study Flow chart

One participant in each group dropped out because of maternity leave. Twenty-one participants were included in the final analysis set for the primary outcome. All but one participant completed the post-program competencies self-assessment, whereas 9 participants in the intervention group and 11 participants in the control group completed the blinded expert assessment. Figure 1 shows the study flow chart.

### Primary outcome

The proportion of participants obtaining at least 25 supervised examinations at 6 months was significantly higher in the intervention group. Seven participants in the intervention group (70%) versus zero (0%) in control group reached the primary outcome, corresponding to a risk difference of 70% (95% CI, 42 to 98%) and a number needed to supervise of 1.4 (95% CI, 1.0 to 2.4), *p* < 0.001. The difference remained significant in a worst-case scenario analysis where the drop out was classified as ‘failure’ in intervention group and ‘success’ in control group (risk difference 55% (95% CI, 23% to 88%, *p* = 0.01). Success seemed to be higher in a pre-specified subgroup of participants working mainly in POCUS-favourable clinical rotations, as reported in Table 2.

**Table 2:** Primary outcome by study group

	Intervention (*N* = 10)	Control (*N* = 11)	Risk difference, % (95% CI)	NNS	*p*-value^a^
Primary outcome, *n* (%)	7 (70)	0 (0)	70 (42 to 98)	1.4	0.001
WCS, *n* (%)	7 (64)	1 (11)	55 (23 to 88)	1.8	0.01
Clinical rotation					
Favourable^b^, *n* (%)	3/4 (75)	0/7 (0)	75 (33 to 100)	1.3	0.02
Unfavourable, *n* (%)	4/6 (66)	0/4 (0)	66 (29 to 100)	1.5	0.06

*CI* confidence interval, *NNS* number needed to supervise, *WCS* worst-case scenario (drop out considered as failure in intervention arm and success in control arm)

^a^Fischer exact test

^b^Internal, emergency and intensive care medicine rotations

### POCUS examinations

During the study period a total of 334 supervised POCUS examinations were performed by participants in the intervention group versus 6 examinations in the control group. The median number was significantly higher in the intervention group (27, interquartile range, IQR: 6 to 44) than in the control group (0, IQR 0 to 0; *p* < 0.001). About half of the total examinations in the intervention group focused on the lungs and were justified by diagnostic purposes, as reported in Table 3.

**Table 3:** Median number and characteristics of POCUS examinations by study group

	Intervention (*N* = 334)	Control (*N* = 6)	*p*-Value
Number of POCUS examinations, median (IQR)			
Total	27 (6 to 44)	0 (0 to 0)	< 0.001
Clinical rotation			
Favourable^a^	39 (16 to 73)	0 (0 to 0)	< 0.001
Unfavourable	28 (24 to 30)	0 (0 to 0)	< 0.001
Investigation			
Lung	13 (3 to 16)	0 (0 to 0)	< 0.001
Heart	14 (3 to 28)	0 (0 to 0)	< 0.001
Reason for POCUS, *n* (%)			
Diagnostic purpose	154 (46%)	6 (100%)	
Training	180 (54%)	0 (0%)	
Type of supervision, *n* (%)			
In person (direct)	201 (60%)	4 (67%)	
Asynchronous (indirect)	133 (40%)	2 (33%)	

*IQR* interquartile range, *POCUS* point of care ultrasonography

^a^Internal, Emergency and Intensive medicine rounds were considered as favourable clinical rotations

### Self-assessment questionary

**Fig. 2:**
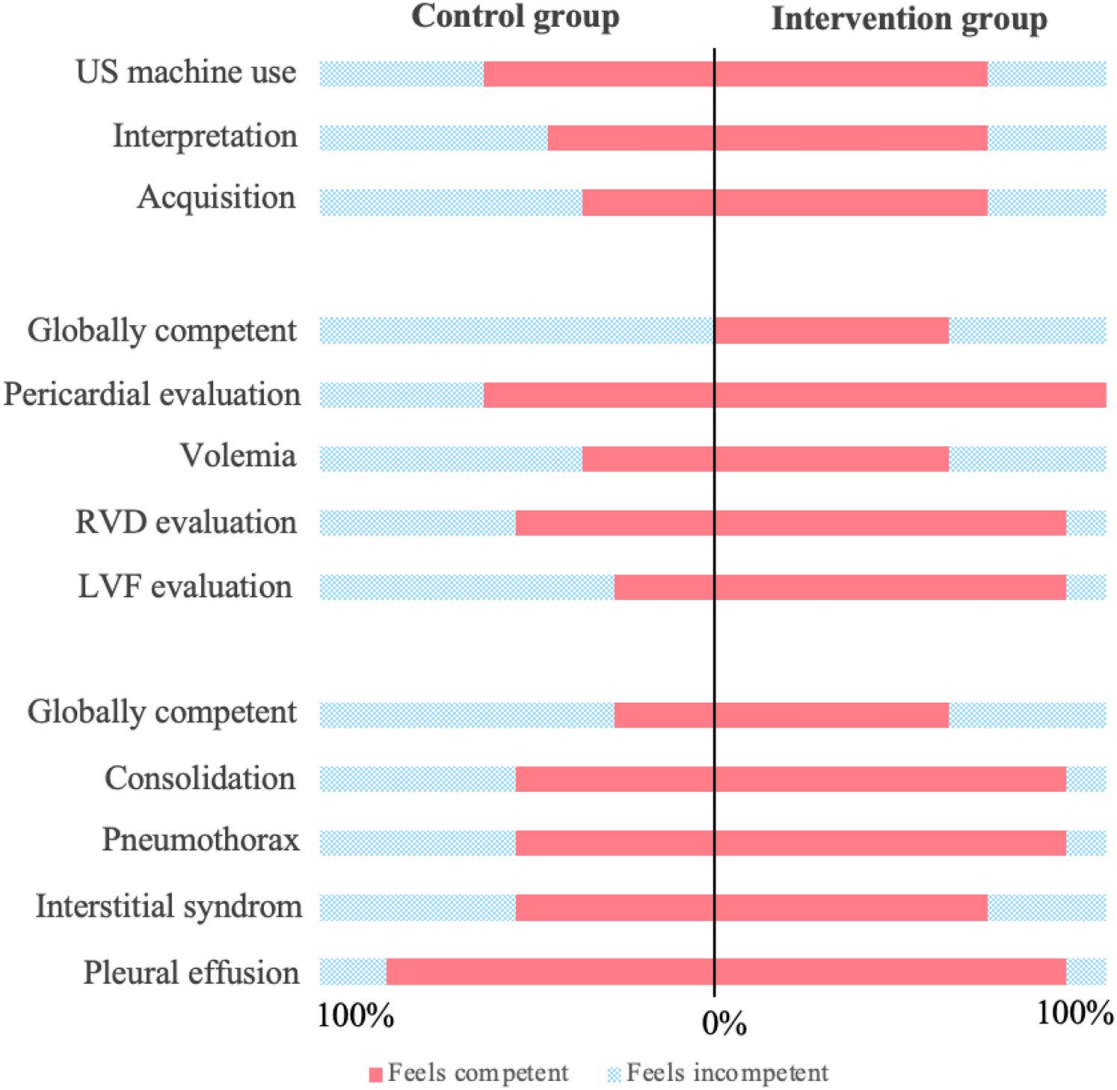
Self-assessed competence in POCUS by study group

At follow-up, a higher proportion of participants in the intervention arm considered themselves globally competent for heart (60% versus 0%, *p* = 0.003) and lung US (60% versus 25%, *p* = 0.19), although the difference was statistically significant only for heart US. Between-group differences were particularly marked for heart applications (e.g. 90% versus 25% of participants considered themselves competent in LVF evaluation, *p* = 0.004). Notably, compared to the pre-tutoring assessment, the intervention group showed an increased proportion of participants who perceived themselves as competent across all items, whereas the control group showed stability or even a decrease in the perceived competencies. Detailed results are reported in Fig. 2 and in Supplementary Table 4.

### Expert testing

**Fig. 3:**
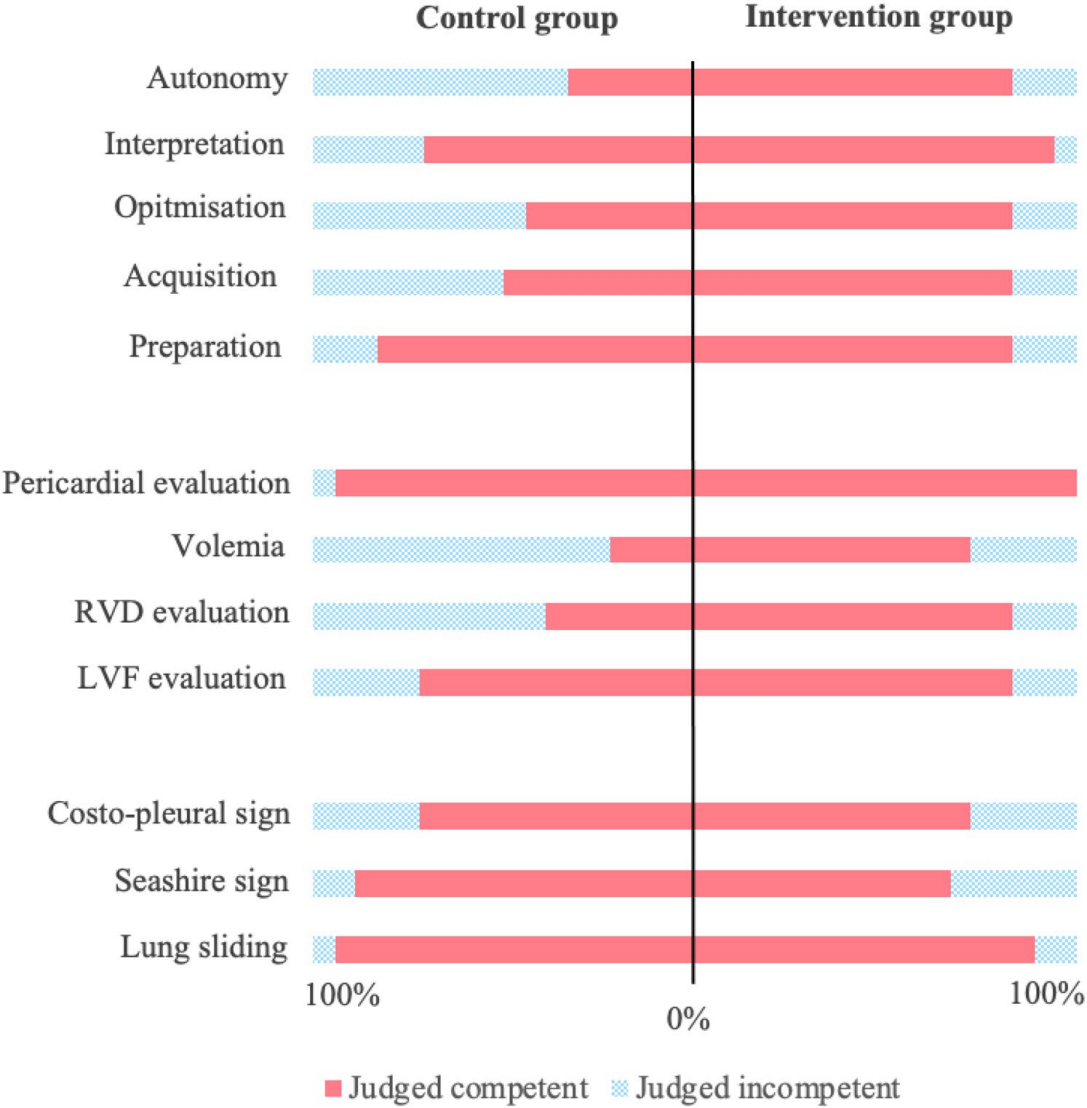
Expert-assessed competence in POCUS by study group

When evaluated by blinded external POCUS experts, a higher proportion of participants in the intervention group were considered autonomous (83% versus 33%, *p* = 0.002), competent for the acquisition of images (83% versus 50%, *p* = 0.034) and for the optimisation of images (83% versus 44% *p* = 0.034). The proportion of participants obtaining high quality images was higher in the intervention group for all focused echocardiographic views, with a difference reaching statistical significance for apical five-chamber (65% versus 11%, *p* = 0.001), subcostal four-chamber (78 versus 44%, *p* = 0.04) and subcostal inferior vena cava views (78% versus 26%, *p* = 0.001). Furthermore, a higher proportion of participants obtained images allowing a meaningful interpretation of RVD (83% versus 39%, *p* = 0.006), and volemia (72% versus 22%, *p* = 0.003). There was no significant difference observed when testing for lung US. Complete results are reported in Fig. 3 and supplementary Table 5.

## Discussion

In this randomized controlled trial following a hands-on heart and lung POCUS course, we tested a 6-month structured POCUS tutoring program versus usual practice in internal medicine residents. In the intervention arm, there was a significant increase in the proportion of residents obtaining at least 25 supervised examinations and in the absolute number of supervised POCUS examinations. This trial succeeds to prove the feasibility and superiority of a tutoring program for POCUS supervision in comparison to the standard of practice in our and many institutions. Whereas participants in control group were strongly encouraged to seek supervision, only a few of them did so. Indeed, low rate of practice is usually observed after practical POCUS courses, as reported in a previous study in which only 6% of participants performed 30 examinations six months after the course [9].

Interestingly, the increased number of prompted examinations appears to enhance perceived and observed proficiency in heart but not lung US. In contrast to our observations concerning the skills in lung US, a previously published study including 28 internal medicine residents, randomized to a standard POCUS curriculum (i.e. a half-day theoretical and practical course followed by 5 one-hour lecture and hands-on sessions) versus 20 additional hours of bedside supervision focused on lung US has demonstrated a significant increase in proficiency in the intervention group after 6 months [15]. Divergence in results it is probably related to the level of assessed competency complexity: participants were asked to demonstrate 23 different lung US competencies, whereas only three simple lung competencies were tested by our assessment tool and were reached by most participants in both groups, even in the complete absence of supervised training after the course. The benefit of a structured supervision may thus be particularly important for the acquisition of complex POCUS skills. Considering the maintenance of complex POCUS skills, our observation of the loss in the perceived global competencies regarding heart ultrasound in the control group (Supplementary Table 4) was also observed in previous studies. In a prospective cohort study including 23 medical students trained in POCUS examination (didactic lectures and hands-on practice on healthy volunteers), the authors reported a decline in image acquisition skills for heart (i.e. parasternal short and long axis, IVC) but not lung US (i.e. lung sliding) [8] Another randomized controlled trial involving 21 critical care fellows failed to demonstrate a statistically significant rise in 6-month POCUS knowledge and skills following 8-h of refresh training, incorporating 6 h of scheduled bedside supervision (assessment tool ranging from 0 to 200 points, higher value indicating greater competence). It is worth noting that all participants were enrolled in a fellowship POCUS curriculum, which encompassed learning modules, in-class lectures, and hands-on training, resulting in an increase in competency scores for all participants. This increase was more pronounced in the intervention group but the between-group difference did not reach statistical significance, possibly due to insufficient statistical power (median increase of 18 (IQR, 3.8 to 38) versus 31 (IQR, 21 to 46) in control and intervention group respectively, *p* = 0.09).[16].

Critical care physicians were the pioneers in POCUS implementation and previous studies focused mostly on critical care fellows [16, 17]. Our study is notable for implementing POCUS tutoring during internal medicine residency, a setting historically known to have lower levels of familiarity with POCUS. The widespread availability of devices in internal medicine at our institution, however, exposed the trainees to a favourable environment for ultrasound utilisation supporting the acquisition of skills. In our subgroup analysis, we observed an incremental benefit when residents were in a clinical rotation with higher availability of US devices (i.e. emergency, critical care and internal medicine). Ultrasonography devices alone without supervision slots are however insufficient, as demonstrated by the extremely low rate of POCUS examinations in control group, despite a greater proportion of participants with favourable rotations. Two previous trials of internal medicine residents randomized to receive personal handheld US devices or not, observed no differences in skills acquisition [18, 19] Consequently, to facilitate skills acquisition in POCUS and to maintain proficiency, in addition to basic training, students should benefit from structured supervision in an environment conducive to the application of this knowledge and equipped with the necessary tools for this task.

Our study has some limitations. First, the study was powered only for the primary outcome and secondary outcomes must be considered as exploratory, due to the small sample size. Second, supervision time slots were scheduled on top of an already demanding clinical workload; unavailability of tutors or trainees may have influenced the success rate notably in control group where no scheduled session were planned in advance. Clinical POCUS rotations with dedicated time has been suggested as a valuable solution and must be explored in further trials [20]. Third, both groups experienced drop-outs due to maternity leave, reflecting the pragmatic design of the trial. However, results maintained statistical significance in our worst-case scenario sensitivity analysis.

Our study has several strengths. First, while the use of POCUS in internal medicine is experiencing exponential growth, there are few studies addressing the challenge of achieving and maintaining proficiency. This study contributes to the subject with high quality data. Second, participants underwent a rigorous and comprehensive learning process, overseen by a team of supervisors, all certified by the Swiss Society of Ultrasound in Medicine (https://sgum-ssum.ch/, Supplementary Table 3). Third, in contrast to prior studies, external POCUS experts were involved in the study to maintain assessor blinding and mitigate the risk of bias, thereby reinforcing the robustness of our assumptions.

In conclusion, our findings highlight the feasibility and need for a structured supervision following a hands-on course to improve both the use and proficiency of POCUS, especially for complex tasks and POCUS applications requiring advanced visuospatial skills, such as focused echocardiography.

The low level of supervised examinations and competency in the control group raises serious doubts regarding the relevance of POCUS single courses without further supervision. Structured POCUS tutoring can significantly enhance training and practice, providing a solid foundation for a practical and safe use of POCUS. Dedicated supervision time slots for trainees during acute medicine clinical rounds may further increase the success rate.

## Supplementary Information

Below is the link to the electronic supplementary material.

Supplementary file1 (DOCX 101 KB)

## References

[1] Leidi A, Rouyer F, Marti C, Reny JL, Grosgurin O (2020) Point of care ultrasonography from the emergency department to the internal medicine ward: current trends and perspectives. Intern Emerg Med. 10.1007/s11739-020-02284-5 32034674 10.1007/s11739-020-02284-5

[2] Guidelines U (2017) Emergency, point-of-care and clinical ultrasound guidelines in medicine. Ann Emerg Med 69(5):e27–e54 28442101 10.1016/j.annemergmed.2016.08.457

[3] Moore CL, Copel JA (2011) Point-of-care ultrasonography. N Engl J Med 364(8):749–757 21345104 10.1056/NEJMra0909487

[4] Ma IWY, Arishenkoff S, Wiseman J, Desy J, Ailon J, Martin L et al (2017) Internal medicine point-of-care ultrasound curriculum: consensus recommendations from the canadian internal medicine ultrasound (CIMUS) group. J Gen Intern Med 32(9):1052–1057 28497416 10.1007/s11606-017-4071-5 PMC5570740

[5] Torres-Macho J, Aro T, Bruckner I, Cogliati C, Gilja OH, Gurghean A et al (2019) Point-of-care ultrasound in internal medicine: a position paper by the ultrasound working group of the European federation of internal medicine. Eur J Intern Med. 10.1016/j.ejim.2019.11.016 31836177 10.1016/j.ejim.2019.11.016

[6] Turner EE, Fox JC, Rosen M, Allen A, Rosen S, Anderson C (2015) Implementation and assessment of a curriculum for bedside ultrasound training. J Ultrasound Med 34(5):823–828 25911715 10.7863/ultra.34.5.823

[7] Kimura BJ, Sliman SM, Waalen J, Amundson SA, Shaw DJ (2016) Retention of ultrasound skills and training in “point-of-care” cardiac ultrasound. J Am Soc Echocardiogr 29(10):992–997 27372559 10.1016/j.echo.2016.05.013

[8] Rappaport CA, McConomy BC, Arnold NR, Vose AT, Schmidt GA, Nassar B (2019) A prospective analysis of motor and cognitive skill retention in novice learners of point of care ultrasound. Crit Care Med 47(12):e948–e952 31569139 10.1097/CCM.0000000000004002

[9] Rajamani A, Miu M, Huang S, Elbourne-Binns H, Pracher F, Gunawan S et al (2019) Impact of critical care point-of-care ultrasound short-courses on trainee competence. Crit Care Med 47(9):e782–e784 31162194 10.1097/CCM.0000000000003867

[10] Wong J, Montague S, Wallace P, Negishi K, Liteplo A, Ringrose J et al (2020) Barriers to learning and using point-of-care ultrasound: a survey of practicing internists in six north American institutions. Ultrasound J 12(1):19 32307598 10.1186/s13089-020-00167-6 PMC7167384

[11] Olgers TJ, Azizi N, Bouma HR, Ter Maaten JC (2020) Life after a point-of-care ultrasound course: setting up the right conditions! Ultrasound J 12(1):43 32893335 10.1186/s13089-020-00190-7 PMC7475055

[12] Finn EM, Zwemer EK, Stephens JR, Dancel R (2023) The state of internal medicine point-of-care ultrasound (POCUS) fellowships in the United States and Canada. Am J Med 136(8):830–836 37116671 10.1016/j.amjmed.2023.04.011

[13] Bell C, Wagner N, Hall A, Newbigging J, Rang L, McKaigney C (2021) The ultrasound competency assessment tool for four-view cardiac POCUS. Ultrasound J 13(1):42 34570287 10.1186/s13089-021-00237-3 PMC8476706

[14] Millington SJ, Arntfield RT, Guo RJ, Koenig S, Kory P, Noble V et al (2017) The assessment of competency in thoracic sonography (ACTS) scale: validation of a tool for point-of-care ultrasound. Crit Ultrasound J 9(1):25 29168030 10.1186/s13089-017-0081-0 PMC5700015

[15] Matthews L, Contino K, Nussbaum C, Hunter K, Schorr C, Puri N (2020) Skill retention with ultrasound curricula. PLoS ONE 15(12):e0243086 33270718 10.1371/journal.pone.0243086 PMC7714199

[16] Suzuki R, Kanai M, Oya K, Harada Y, Horie R, Sekiguchi H (2022) A prospective randomized study to compare standard versus intensive training strategies on long-term improvement in critical care ultrasonography proficiency. BMC Med Educ 22(1):732 36280812 10.1186/s12909-022-03780-2 PMC9594969

[17] Beraud AS, Rizk NW, Pearl RG, Liang DH, Patterson AJ (2013) Focused transthoracic echocardiography during critical care medicine training: curriculum implementation and evaluation of proficiency*. Crit Care Med 41(8):e179–e181 23760156 10.1097/CCM.0b013e31828e9240

[18] Buesing J, Weng Y, Kugler J, Wang L, Blaha O, Hom J et al (2021) Handheld ultrasound device usage and image acquisition ability among internal medicine trainees: a randomized trial. J Grad Med Educ 13(1):76–82 33680304 10.4300/JGME-D-20-00355.1 PMC7901629

[19] Kumar A, Weng Y, Wang L, Bentley J, Almli M, Hom J et al (2020) Portable ultrasound device usage and learning outcomes among internal medicine trainees: a parallel-group randomized trial. J Hosp Med 15(2):e1–e6 32118565 10.12788/jhm.3351

[20] Hayward M, Chan T, Healey A (2015) Dedicated time for deliberate practice: one emergency medicine program’s approach to point-of-care ultrasound (PoCUS) training. CJEM 17(5):558–561 26030268 10.1017/cem.2015.24

