# Supplementary material for: Heart and lung point-of-care ultrasonography tutoring in internal medicine: a randomized controlled trial
Source: J Ultrasound. 2024 Dec 3;28(1):1–9. doi: 10.1007/s40477-024-00968-8 (PMC11947348; doi:10.1007/s40477-024-00968-8)
Supplement: Supplementary file 1 — Supplementary file1 (DOCX 101 KB) [file 40477_2024_968_MOESM1_ESM.docx]

**Heart and Lung Point-of-Care Ultrasonography Tutoring in Internal Medicine: a Randomized Controlled Trial**

**Supplementary materials**

Submission to Journal of Ultrasound

**Supplementary Figure 1. Trial design**

**
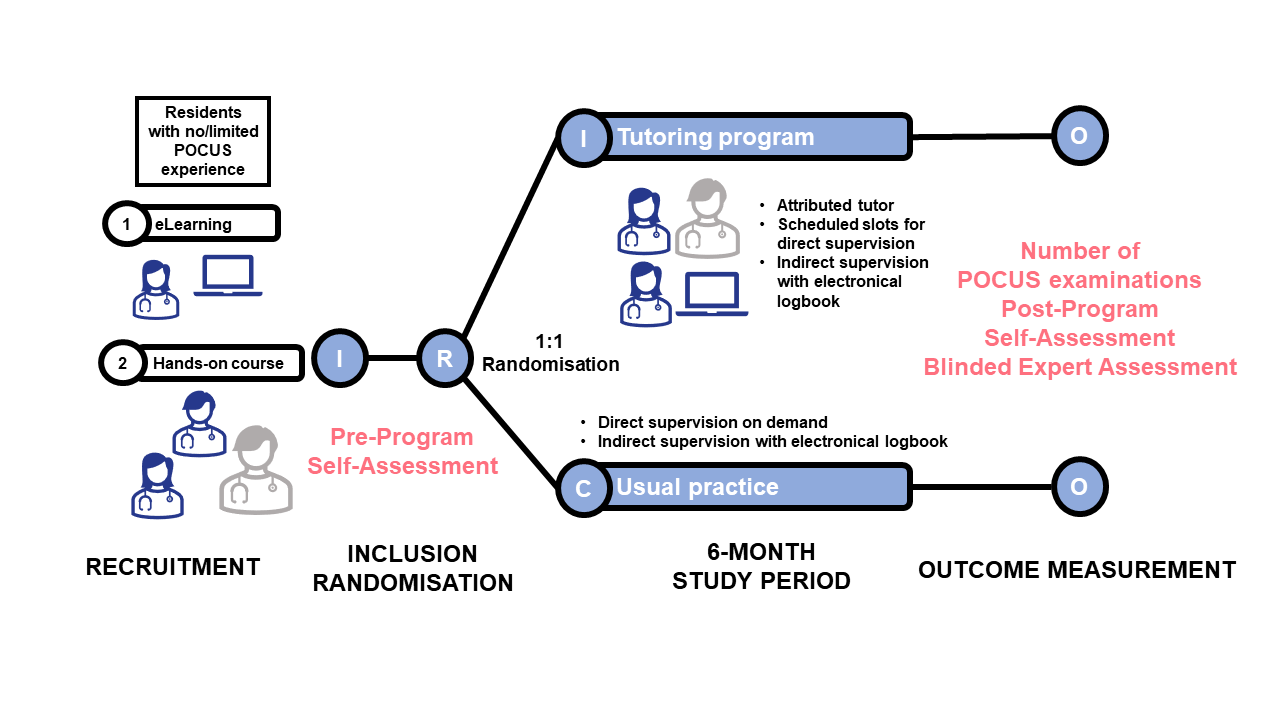
**

**Supplementary Table 1. Self-assessment questionary**

*General skills and machine operations*

|  | Strongly  disagree | Disagree | Neutral | Agree | Strongly agree |
| --- | --- | --- | --- | --- | --- |
| 1. I am confident in general image acquisition skills 2. I am confident in general image interpretation skills 3. I am confident in machine operations | 1  1  1 | 2  2  2 | 3    3  3 | 4  4  4 | 5  5  5 |

*Cardiac ultrasound*

| 1. I am confident in evaluation skills for cardiac systolic function | 1 | 2 | 3 | 4 | 5 |
| --- | --- | --- | --- | --- | --- |
| 1. I am confident in evaluation skills for heart chambers size (RV/LV) 2. I am confident in evaluation skills for inferior vena cava 3. I am confident in evaluation skills for pericardial effusion | 1  1  1 | 2  2  2 | 3  3  3 | 4  4  4 | 5  5  5 |

*Pulmonary ultrasound*

| 1. I am confident in evaluation skills for pleural effusion | 1 | 2 | 3 | 4 | 5 |
| --- | --- | --- | --- | --- | --- |
| 1. I am confident in evaluation skills for interstitial syndrome 2. I am confident in evaluation skills for pneumothorax 3. I am confident in evaluation skills for consolidation | 1  1  1 | 2  2  2 | 3  3  3 | 4  4  4 | 5  5  5 |
|  |  |  |  |  |  |
|  |  |  |  |  |  |

**Supplementary Table 2. Expert assessment tool**

Generals skills

|  | Skills acquired for **CERTAIN** criteria | Skills acquired for **MOST** criteria | Skills acquired for **ALL** criteria |
| --- | --- | --- | --- |
| **Preparation**  - Patient & scanner positioning  - Probe selection  - Initial pre-settings (choosing the right mode) | 1 | 2 | 3 |
| **Image acquisition**  - Positioning the probe in the hand  - Identification of anatomical landmarks  - Smooth movements  - Appropriate measurements | 1 | 2 | 3 |
| **Image optimization**  - Area of interest centering  - Overall image quality  - Optimization of settings (depth, gain) | 1 | 2 | 3 |
| **Clinical integration**  - Appropriate interpretation | 1 | 2 | 3 |

|  | I have to do the exam | Physical intervention would be required (probe repositioning) | Verbal intervention would be necessary | Back-up presence required | I don't need to be there |
| --- | --- | --- | --- | --- | --- |
| **Autonomy** | 1 | 2 | 3 | 4 | 5 |

Skills scale for pleuropulmonary ultrasonography

**Image acquisition**

*Please note the image quality for each view according to the scale below:*

|  | Not obtained | Insufficient quality for interpretation |  | Suboptimal quality, but interpretation possible |  | Good quality and easy to interpret |
| --- | --- | --- | --- | --- | --- | --- |
| **Pleural sliding** | 0 | 1 | 2 | 3 | 4 | 5 |
| **Beach sign** | 0 | 1 | 2 | 3 | 4 | 5 |
| **Costo-diaphragmatic recess** | 0 | 1 | 2 | 3 | 4 | 5 |

Cardiac ultrasound competency scale

**Image acquisition**

*Please rate the image quality for each view according to the scale below:*

|  | Not obtained | Insufficient quality for interpretation |  | Suboptimal quality, but interpretation possible |  | Good quality and easy to interpret |
| --- | --- | --- | --- | --- | --- | --- |
| **Parasternal long axis view**  - Setpum as horizontal as possible  - Apex not visible  - Aortic and mitral valves visible  - Descending aorta visible | 0 | 1 | 2 | 3 | 4 | 5 |
| **Parasternal short axis view**  - Round shape of the LV  - Papillary muscles visible  - RV visible | 0 | 1 | 2 | 3 | 4 | 5 |
| **Apical four-chamber view**  - 4 cavities fully visible  - Tricuspid and mitral valves visible  - Septum vertical and in the center of the image  - TAPSE measurement | 0 | 1 | 2 | 3 | 4 | 5 |
| **Apical five-chamber view**  - Visible aortic valve | 0 | 1 | 2 | 3 | 4 | 5 |
| **Subcostal view**  - 4 cavities visible  - Tricuspid and mitral valves visible | 0 | 1 | 2 | 3 | 4 | 5 |
| **Inferior vena cava**  - Opening into RA  - As horizontal as possible  - Inspiro-expiratory diameter measurement | 0 | 1 | 2 | 3 | 4 | 5 |

RA : right auricule

**Image interpretation**

*On the basis of the images produced, is it possible to interpret :*

|  | Image quality does not allow interpretation | Image quality enables interpretation |
| --- | --- | --- |
| **LV function** | 0 | 1 |
| **Presence/absence of pericardial effusion** | 0 | 1 |
| **Presence/absence of RVD** | 0 | 1 |
| **Presence/absence of hypo/hypervolaemia** | 0 | 1 |

RVD: right ventricular dilatation

*What is the most likely diagnosis? (check the candidate's answer)*

| **Normal LVF** |  |
| --- | --- |
| **Moderately reduced LVF** |  |
| **Severely impaired LVF** |  |
| **Pericardial effusion** |  |

LVF : lleft ventricular systolic function

**Supplementary Table 3. Characteristics of POCUS tutors**

|  | Tutor 1 | Tutor 2 | Tutor 3 |
| --- | --- | --- | --- |
| POCUS certification, y | 2018 | 2019 | 2022 |
| Seniority | Attending physician | Chief resident | Chief resident |
| Supervised POCUS, n (%) | 180 (53%) | 83 (24%) | 70 (23%) |
| Trainees reaching the primary outcome (%) | 100% | 75% | 25% |

**Supplementary Table 4. Pre- and post-tutoring self-assessment questionary**

| **Participants** | **Pre-tutoring**  **(N=23)** | **Post-tutoring**  **(N=22)** | | **p-Value^a^** |
| --- | --- | --- | --- | --- |
|  | **All subjects**  **(N=23)** | **Intervention**  **(N=10)** | **Control**  **(N=12)** |  |
| **General** |  |  |  |  |
| Acquisition, n (%)  Competent  Incompetent | 9 (39)  14 (61) | 7 (70)  3 (30) | 4 (33)  8 (67) | 0.08 |
| Interpretation, n (%)  Competent  Incompetent | 9 (39)  14 (61) | 7 (70)  3 (30) | 5 (42)  7 (58) | 0.2 |
| US machine use, n (%)  Competent  Incompetent | 12 (52)  11 (48) | 7 (70)  3 (30) | 7 (58)  5 (42) | 0.7 |
| **Echocardiography** |  |  |  |  |
| LVF evaluation, n (%)  Competent  Incompetent | 14 (61)  9 (39) | 9 (90)  1 (10) | 3 (25)  9 (75) | 0.004* |
| RVD evaluation, n (%)  Competent  Incompetent | 10 (43)  13 (57) | 9 (90)  1 (10) | 6 (50)  6 (50) | 0.07 |
| Volemia, n (%)  Competent  Incompetent | 8 (35)  15 (65) | 6 (60)  4 (40) | 4 (33)  8 (67) | 0.4 |
| Pericardial evaluation, n (%)  Competent  Incompetent | 17 (74)  6 (26) | 10 (100)  0 (0) | 7 (58)  5 (42) | 0.02* |
| Globally competent, n (%)  Yes  No | 7 (30)  16 (70) | 6 (60)  4 (40) | 0 (0)  12 (100) | 0.003* |
| **Lung ultrasonography** |  |  |  |  |
| Pleural effusion  Competent  Incompetent | 19 (83)  4 (17) | 9 (90)  1 (10) | 10 (83)  2 (17) | 1.0 |
| Interstitial syndrome  Competent  Incompetent | 16 (70)  7 (30) | 7 (70)  3 (30) | 6 (50)  6 (50) | 0.4 |
| Pneumothorax  Competent Incompetent | 13 (57)  10 (43) | 9 (90)  1 (10) | 6 (50)  6 (50) | 0.07 |
| Consolidation  Competent  Incompetent | 12 (48)  11 (52) | 9 (90)  1 (10) | 6 (50)  6 (50) | 0.07 |
| Globally competent, n (%)  Yes  No | 6 (26)  17 (74) | 60 (60)  40 (40) | 3 (25)  9 (75) | 0.19 |

US: ultrasonography; IVC inferior vena cava; LVF: left ventricular systolic dysfunction; RVD: right ventricular dilatation

**Supplementary Table 5. Blinded-expert assessment, by study group**

| **Participants** | **Intervention**  **(N obs=18)** | **Control**  **(N obs=18)** | **p-Value^a^** |
| --- | --- | --- | --- |
| **General** |  |  |  |
| Preparation, n (%)  Competent  Incompetent | 15 (83)  3 (17) | 15 (83)  3 (17) | 1.0 |
| Acquisition, n (%)  Competent  Incompetent | 15 (83)  3 (17) | 9 (50)  9 (50) | 0.034* |
| Optimisation, n (%)  Competent  Incompetent | 15 (83)  3 (17) | 8 (44)  10 (56) | 0.015* |
| Interpretation, n (%)  Competent  Incompetent | 16 (94)  1 (6) | 10 (71)  4 (29) | 0.14 |
| Autonomy, n (%)  Yes  No | 15 (83)  3 (17) | 6 (33)  12 (67) | 0.002* |
| **Echocardiography** |  |  |  |
| Quality of acquisition |  |  |  |
| PSLA, n (%)  High  Poor | 15 (83)  3 (17) | 11 (61)  7 (39) | 0.14 |
| PSSA, n (%)  High  Poor | 13 (72)  5 (28) | 9 (50)  9 (50) | 0.17 |
| 4C, n (%)  High  Poor | 13 (72)  5 (28) | 8 (44)  10 (56) | 0.09 |
| 5C, n (%)  High  Poor | 11 (65)  6 (35) | 2 (11)  16 (89) | 0.001* |
| SC, n (%)  High  Poor | 14 (78)  4 (22) | 8 (44)  10 (55) | 0.04* |
| IVC, n (%)  High  Poor | 14 (78)  4 (22) | 3 (26)  14 (76) | 0.001* |
| Meaningful interpretation |  |  |  |
| LVF evaluation, n (%)  Possible  Impossible | 15 (83)  3 (17) | 13 (72)  5 (28) | 0.69 |
| RVD evaluation, n (%)  Possible  Impossible | 15 (83)  3 (17) | 7 (39)  11 (61) | 0.006* |
| Volemia, n (%)  Possible  Impossible | 13 (72)  5 (28) | 4 (22)  14 (78) | 0.003* |
| Pericardial evaluation, n (%)  Possible  Impossible | 18 (100)  0 (0) | 17 (94)  1 (6) | 1.0 |
| Proportion of correct LVF evaluation, n (%)  Total  Patients  Healthy Volunteers | 17 (94)  8 (89)  9 (100) | 14 (82)  5 (62)  9 (100) | 0.34  0.29  1.0 |
| **Lung ultrasonography** |  |  |  |
| Quality of acquisition |  |  |  |
| Lung sliding, n (%)  High  Poor | 16 (89)  2 (11) | 17 (94)  1 (6) | 1.0 |
| Seashore sign, n (%)  High  Poor | 12 (67)  6 (33) | 16 (89)  2 (11) | 0.23 |
| Costo-pleural space, n (%)  High  Poor | 13 (72)  5 (27) | 13 (72)  5 (27) | 1.0 |

PSLA: parasternal long axis view; PSSA: parasternal short axis view; 4C: apical four-chamber view; 5C: apical five-chamber view; SC: subcostal view; IVC inferior vena cava; LVF: left ventricular systolic dysfunction; RVD: right ventricular dilatation
